# Organoid and Spheroid Tumor Models: Techniques and Applications

**DOI:** 10.3390/cancers13040874

**Published:** 2021-02-19

**Authors:** Sreenivasulu Gunti, Austin T.K. Hoke, Kenny P. Vu, Nyall R. London

**Affiliations:** 1 Sinonasal and Skull Base Tumor Program, Head and Neck Surgery Branch, National Institute on Deafness and Other Communication Disorders, National Institutes of Health, Bethesda, MD 20892, USA; guntis@nih.gov (S.G.); austin.hoke@nih.gov (A.T.K.H.); kenny.vu@nih.gov (K.P.V.); 2 University of North Carolina at Chapel Hill—School of Medicine, Chapel Hill, NC 27516, USA; 3 Department of Otolaryngology—Head and Neck Surgery, Johns Hopkins University, Baltimore, MD 21218, USA; 4 Department of Neurosurgery, Johns Hopkins University, Baltimore, MD 21218, USA

**Keywords:** organoids, spheroids, air–liquid interface, personalized medicine, drug screening, tumor modeling, organoid biobanks, immunotherapy, microfluidics, organoids-on-chip

## Abstract

**Simple Summary:**

Cell cultures can be carried out in three dimensions (3D). Organoids and spheroids are different 3D cell culture models that can be cultured with different techniques. These 3D cell culture units established from a patient tumor have several similarities to the original tumor tissue and possess several advantages in conducting basic and clinical cancer research. Organoids prepared from a patient tissue can be preserved in a living biobank. Testing chemo-, radio- and immuno-therapies on these organoids has the potential to predict the patient responses and these models have incredible promise for personalized medicine. This review presents different organoid models, the techniques to prepare them and recent advances in their applications.

**Abstract:**

Techniques to develop three-dimensional cell culture models are rapidly expanding to bridge the gap between conventional cell culture and animal models. Organoid and spheroid cultures have distinct and overlapping purposes and differ in cellular sources and protocol for establishment. Spheroids are of lower complexity structurally but are simple and popular models for drug screening. Organoids histologically and genetically resemble the original tumor from which they were derived. Ease of generation, ability for long-term culture and cryopreservation make organoids suitable for a wide range of applications. Organoids-on-chip models combine organoid methods with powerful designing and fabrication of micro-chip technology. Organoid-chip models can emulate the dynamic microenvironment of tumor pathophysiology as well as tissue–tissue interactions. In this review, we outline different tumor spheroid and organoid models and techniques to establish them. We also discuss the recent advances and applications of tumor organoids with an emphasis on tumor modeling, drug screening, personalized medicine and immunotherapy.

## 1. Introduction

Cancer represents the second most leading cause of death globally, accounting for one in six deaths according to the World Health Organization (WHO) [1]. In the past decade, significant advances have been made in cancer research in terms of diagnosis and treatment. However, a large proportion of drugs fail at the development stage and only a few drugs reach the market for clinical use. The high attrition rates are in part due to failure to meet safety requirements or from lack of efficacy in clinical trials [2]. For example, a drug that appears to be safe in animal models can show unacceptable toxicities in humans, thus leading to withdrawal. One of the major obstacles in developing drugs in a time- and cost-effective manner is the lack of preclinical cancer models that resemble the complexity of human tumors. Two-dimensional (2D) cell culture has significantly advanced several areas of research and remains a predominant pre-clinical method [3]. Cell culture methods are relatively simple, economic and are amenable to high-throughput drug screening and toxicity studies. However, 2D cell cultures are oversimplified versions of tumors, and do not recapitulate all the essential cellular organization and interactions that occur in vivo. The success rate of establishment of cell lines for some cancer types is very low. Moreover, cell lines often lose cellular heterogeneity observed in tumors upon long-term culture. On the other hand, patient-derived tumor xenografts (PDTX) resemble the tumor in cellular complexity and can retain tumor heterogeneity. PDTXs are often implanted in immunodeficient hosts and thus require reconstitution of autologous immune cells if immunity is studied. The low implantation-take rates, duration of immune reconstitution, cost and time to generate humanized models are major limitations of PDTXs [4] (Table 1). However, cancer is a highly heterogenous disease and the tumor microenvironment (TME) is complex and dynamic. Both cellular (tumor epithelium, fibroblasts, stem cells, endothelial cells and tumor-infiltrating immune cells) and non-cellular components (extra cellular matrix (ECM), cytokines, chemokines and growth factors) of the TME play a crucial role in tumor development and progression and thus could play a critical role in the outcome of drug development [5]. Therefore, pre-clinical models that recapitulate tumor pathophysiology in vivo are critical for the accurate assessment of drug efficacy and toxicity.

Three-dimensional cell culture techniques have emerged as a promising method to bridge the gap between cell culture and animal models. Various 3D models have been developed, including tissue explants, spheroids and the recently expanding field of organoids. The terms organoids, spheroids and 3D cell cultures have been used interchangeably in the literature [6]. Spheroids are spherical cellular units that are generally cultured as free-floating aggregates and are arguably of low complexity in mirroring tumor organization. In general, organoids can be referred to as cells grown in 3D to form structural units that partially resemble the organ, both in structure and function [7]. 3D cultures can be established either using support of an ECM (scaffold-based) or without the use of a scaffold (scaffold-free). Spheroids and organoid culture models have distinct and overlapping purposes and they differ in terms of tumor cell sources, protocol for culture and the time required for establishment (Table 2). Organoids can be expanded for long-term culture and can be cryopreserved. Organoids resemble the original tissue both histologically and genetically. In addition, organoids can be cultured from a very small amount of tissue and are amenable to genetic manipulations [8,9]. These features allow their use for a wide range of applications in cancer research, including the study of carcinogenesis, drug development and personalized medicine. In this review, we highlight different models of spheroids and organoids based on their cellular sources and methods of generation, including static and microfluidic flow-based chip methods. We focus on recent progress in the application of tumor organoids in cancer modeling, drug development, personalized cancer medicine and immunotherapy. Applications of spheroids from different cellular sources have been reviewed extensively elsewhere and hence we mainly focus on tumor-derived organoids. However, we include recent literature on tumor-derived spheroids in respective sections [10,11,12]. Finally, we conclude with merits and challenges of organoid technology in cancer biology.

## 2. Spheroid Models and Methods

Spheroids were first introduced in the early 1970s by Sutherland and colleagues [13]. Since then, different spheroid models and methods to generate them have been developed. Spheroids form by spontaneous aggregation of cells followed by binding of cell surface integrins to the ECM. After initial cell–cell contact, cells upregulate E-Cadherin which accumulates on the cell surface and then the spheroid becomes a compact structure through strong intercellular E-cadherin interactions [14,15]. This process is impacted by various factors including nutrients, oxygen and growth factors [16].

Different spheroid models have been described based on their cellular sources. Multicellular tumor spheroids (MCTS) are often made from cancer cell lines, but rarely from tumor tissues. MCTS show little histological resemblance to the original tumor, but they mimic metabolic and proliferation gradients of the in vivo tumor and model clinically relevant resistance to chemotherapy. The advantages of MCTS are that they are clonal, simple to expand into large cultures and suitable for high-throughput systems [17]. Tumor-derived spheroids are prepared from mechanical or enzymatic dissociation of tumor tissue into a single cell suspension, followed by culture in serum or serum-free media. Tumor-derived spheroids have been prepared from brain [18], breast [19], lung [20], colon [21], prostate [22], pancreas [23] and ovarian tumors [24]. Culture in serum-free media containing different growth factors such as hydrocortisone, insulin and progesterone promotes the growth of tumor cells with stem cell features while selecting against non-malignant and differentiated cells, thus enriching for cancer stem cells. Therefore, a main feature of tumor-derived spheroids is enrichment of cancer stem cells. Organotypic multicellular tumor spheroids are similar to ex vivo explant cultures where the tumor is chopped into 0.3 mm slices or partially dissociated mechanically or enzymatically and cultured in plates coated with agar in serum-containing media [25]. 

Spheroids can be cultured with or without the support of ECM. Scaffold-based methods are generally used in tissue engineering and regenerative medicine applications. Scaffold-free methods are commonly used as they are relatively simple, inexpensive and rapid to generate spheroids. Several different scaffold-free approaches for spheroid formation have been developed. The most simple scaffold-free approach is pellet culture, that involves the centrifugation of cell suspension to concentrate the cells. The centrifugal force helps to promote cell–cell adhesion. This method makes large diameter spheroids and has been used to study chondrogenesis, bone formation and differentiation of mesenchymal stem cells [26,27]. Major limitations to this method include that the shear force by centrifugation can damage the cells as well as the difficulty in large-scale production and visualization of spheroids while they grow.

The hanging drop method is one of the oldest methods and involves the pipetting of 20–40 µL of cell suspension on a lid, which is then inverted, leading to cell aggregation by surface tension and gravitational force. The size of the spheroid can be adjusted by initial cell number and heterotypic spheroids can be formed by co-culturing. Although the method is popular for its simplicity and amenability to high-throughput screening, it is difficult to track spheroid formation and drug perturbations are not directly feasible [28]. Another popular and simple method is liquid overlay. Here, cell suspensions are plated on either low-adhesive surface plates or plates coated with materials such as agar or agarose that prevent cell attachment to the surfaces. Constant rocking of the plate on a shaker promotes cell aggregation. Liquid overlay can be performed in 96-well plates, making it a simple and popular method. Formation of spheroids can be monitored in real-time. One disadvantage of liquid overlay is that the size and shape of the spheroids are not controllable [29]. Spinner cultures and rotating wall vessel cultures rely on constant stirring or rotating respectively, to prevent the settling of cells. Maintaining constant speed is critical in these methods, as too-slow a speed can settle the spheroids and too-high a speed can damage the cells. Due to shear forces, this method cannot be applied to cells with low cohesiveness [30]. 

Tumor spheroids are the simplest of the 3D cell culture models but are popular as they emulate properties of solid tumors in several aspects. Most importantly, they exhibit cell–cell and cell–ECM interactions. In addition, spheroids closely resemble non-vascularized or poorly vascularized tumors and portray metabolic gradients when grown larger than 500 µm. The multilayered structure has an outer layer of proliferating cells, a middle layer of quiescent cells and an inner layer of hypoxic and necrotic cells [31]. These properties of tumor spheroids confer anti-cancer drug resistance as well as resistance to radiation, as seen in human cancers. Hence, tumor spheroids have been widely used in drug screening studies. Moreover, tumor spheroids can be combined with different cell types to use in such applications as cancer cell migration and invasion [32]. 

## 3. Organoid Methods and Models

Organoids can be cultured from embryonic stem cells, induced pluripotent stem cells (PSC) and adult stem cells (ASC). Organoids from these cellular sources form structures through developmental processes and require ECM as a source of basal lamina. Organoids from embryonic stem cells are first expanded and then subsequently differentiated in a multistep protocol which eventually achieves fully differentiated structure [33,34]. The formation of a complex structure generally takes 2 to 3 months depending on the type of tissue and requires a different set of cocktails and growth factors used at different steps [34]. Organoids derived from PSC are structurally complex and recapitulate organ development ex vivo and may contain mesenchymal, epithelial and sometimes endothelial cells as well. Therefore, PSC-derived organoids are an excellent model for studying organogenesis, genetic pathology and infectious diseases, especially for those organs that have little or no regenerative capacity such as the brain [35,36,37,38,39,40]. However, PSC-derived organoid differentiation is not highly efficient at times, for example kidney organoid differentiation has been shown to yield up to 20% unintended cells, including neuronal cells [41]. In addition to longer differentiation times, several PSC-derived organoids retain fetal resemblance and do not recapitulate adult gene expression [33,40]. ASC-derived epithelial organoids model tissue repair processes and thus can only be made from tissue compartments with regenerative capacity. ASC organoids can be derived from normal adult epithelial tissues as well as from malignant tissues and can be expanded long term. ASC-derived organoids are of lower complexity than PSC-derived organoids, however, they recapitulate the histological and genetic features of their parent tissues [8,42]. 

Clevers’ group first developed ASC organoid technology for intestines based on the findings that Lgr5 is a marker for adult gut stem cells which is driven by Wnt-signaling. Single Lgr5+ cells were embedded in laminin-rich basement membrane extract (BME) and cultured into self-organizing crypt villus-like structures by submerging the cells in a cocktail of growth factors [42]. These growth factors, which represent the stem cell niche factors, support the self-renewal of tissue resident stem cells and provide mitogenic stimuli. The composition of stem cell niche factors varies from tissue to tissue. Organoid outgrowth of most epithelial cells requires Wnt activators (Wnt3a and R-spondin), receptor tyrosine kinase ligands (epidermal and fibroblast growth factors), bone morphogenic protein inhibitor noggin and TGF-β inhibitor [43]. Following this submerged approach, organoids for different normal and tumor tissues, including brain [44,45], stomach [36], esophagus [46], lung [47], liver [48], pancreas [49], kidney [50], salivary gland [51], ovary [52], fallopian tube [53], breast [54], colon [55] and prostate [56], have been generated by modifying cell isolation procedures and growth factor cocktails. This submerged method generally represents epithelial-only organoids and does not include stroma [8].

**Figure 1:**
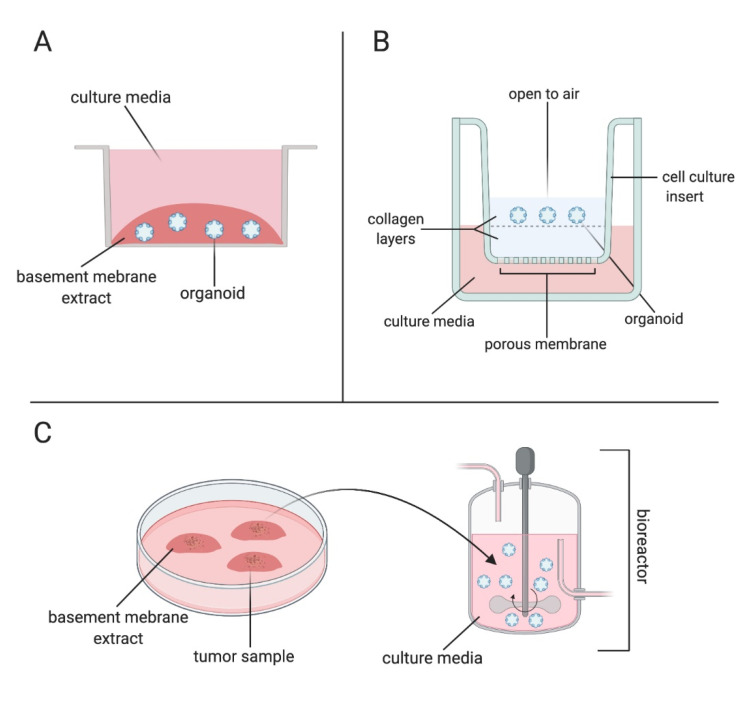
Methods of organoid culture. (**A**) Organoids can be cultured in the submerged method by disrupting tissue mechanically and enzymatically into single-cell suspensions, followed by embedding them in basement membrane extract (BME) and submerging in the culture media. (**B**) In the air–liquid interface (ALI) method, tissue is minced into smaller fragments and embedded in a layer of collagen, which is assembled in a cell culture insert that has a layer of acellular collagen. The cell culture insert is placed in a culture dish with media. (**C**) Alternatively, tissue fragments can be embedded in BME and followed by transferring into a bioreactor.

Similarly, the air–liquid interface (ALI) method was developed, which allows the propagation of organoids both with epithelial and stromal cells [57]. The ALI method utilizes Boyden chambers (cell culture inserts) popularly used for cell migration assays. Cells are embedded in ECM gels in an upper surface of the cell culture inserts with a porous membrane underneath and cells are directly exposed to oxygen, which substantially increases the oxygen supply to the cells compared to an epithelial-only submerged organoid method (Figure 1). Cells obtain nutrients and growth factors from the medium placed in the outer dish through diffusion across the porous membrane on the lower surface. In the ALI method, organoids cultured from neonatal tissues grow without external niche factor supplementation because these factors are thought to be produced from stromal cells within organoids. However, organoids generated from adult tissues require external addition of growth factors. The distinct advantage of the ALI method is that it not only includes stromal cells but can also retain the tumor microenvironment for an extended period of time [58,59].

In another approach, tissue was embedded into droplets of BME and then transferred into spinning bioreactors [60] (Figure 1). The continuous agitation in this method provides better absorption of nutrients and oxygen compared to the two aforementioned static methods [44]. This approach has been used to produce cerebral and retinal organoids [61]. Very recently, glioblastoma organoids were prepared using this similar agitation method, but without mitogens and BME and with a defined culture medium [45]. Interestingly, glioblastoma organoids generated using this method retained histological, genetic features and partial preservation of the microvasculature, as well as immune cells of the original tumor [45]. 

## 4. Microfluidics and Organoid-On-A-Chip Models

Conventional methods of organoid and spheroid culture are static and can lead to accumulation of biochemical waste in the central portion of the organoid, which can be detrimental to cell viability [62]. Although organoids constructed by the ALI method can include epithelial and stromal components and can reconstitute the tumor microenvironment [58], they lack tissue–tissue interfaces and mechanical cues. Generation of organoids by microfluidic technology circumvents some of these limitations. Microfluidic devices are capable of handling microscale volumes of liquids [62]. 

Microfluidic organ-on-a-chip models have recently been developed for a variety of organs, including lung [63,64], liver [65,66], kidney [67] and heart [68], to model these organs in vitro [62]. Organ-on-a-chip models rely on design and engineering principles to precisely control the organ microenvironment, and these synthetic microenvironments are integrated with living cells to mimic organ-level functions in vitro [69]. Additionally, different microfluidic organ-on-a-chip can be interconnected with each other to build body-on-a-chip models which are capable of simulating multi-organ interactions. These micro-physiological systems can be harnessed to study cancer multi-organ metastasis [62]. However, organ-on-a-chip models developed so far utilized either primary cells or cancer cell lines, which cannot resemble the cellular complexity of organs and tumors. The design principles of organ-on-a-chip models can be combined with the self-organization principles of organoids to generate powerful organoid-on-a-chip models. Organ-on-a-chip and organoids are fundamentally different, yet they are complementary models. Hence, organoid-on-a-chip models represent a synergistic combination and could model organ physiology in vitro better than either of them alone [62]. 

**Figure 2:**
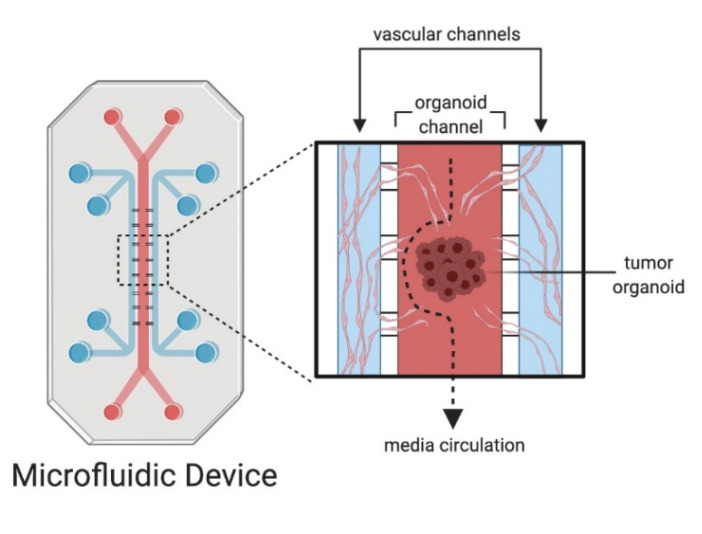
Microfluidic devices and organoids on chips. Microfluidic chips can be designed to culture organoids in a chamber connected to an inlet that can circulate culture media. In a vascular organoid-on-chip model, tumoroids are cultured in a central chamber. Adjacent chambers are connected to the central chamber and endothelial cells with fibroblasts are cultured in hydrogel. The tumoroid cultures are perfused with vasculature and can model angiogenesis.

Several microfluidic chips have been developed for the culture of spheroids to study applications such as drug efficacy testing, drug penetration into tumor and vascularization [62,70,71,72,73]. However, a majority of these studies utilized cancer cell lines to construct spheroids and only few studies used patient-derived tumor tissue [74]. Aref and colleagues utilized commercially available DAX-1 microfluidic devices, which have a central channel for tumor cells surrounded by two channels for media circulation, to culture murine- and patient-derived organotypic tumor spheroids. Interestingly, when the authors cultured tumor spheroids in collagen hydrogels, tumor spheroids reconstituted native immune cells. Further, circulating side channels with anti-PD-1 (programmed cell death protein 1) antibody killed the tumor cells mediated by CD8^+^-T cells, thus demonstrating that patient-derived organotypic tumor spheroids retaining autologous immune cells can be constructed in microfluidic devices [75,76]. Shirure et al. reported the design of a vascularized tumor-on-chip model to emulate physiological mass transport at the arterial end of capillaries within the TME. The fabricated microfluidic chip has a central chamber with perfusable microvasculature in a hydrogel compartment and adjacent chambers for loading tumoroids from cancer cell lines or patient tumor samples [77]. The vascular chamber and tumoroid chambers are interconnected with porous gel through which capillaries outgrew into the tumor chamber from the vascular chamber. The authors have demonstrated the efficient delivery of nutrients and/or drugs to the tumor tissue through the vascular network that helped to maintain tumoroids as physiologically active for long time (Figure 2). The salient features of this vascular tumoroid-on-chip model include the emulation of dynamic tumor evolution by cell proliferation, angiogenesis, migration and intravasation. These tumoroid-on-chip models may pave the way for the modeling of organoids from several cancer types [78].

## 5. Applications of Patient-Derived Organoids and Spheroids

### 5.1. Cancer Modeling

**Figure 3:**
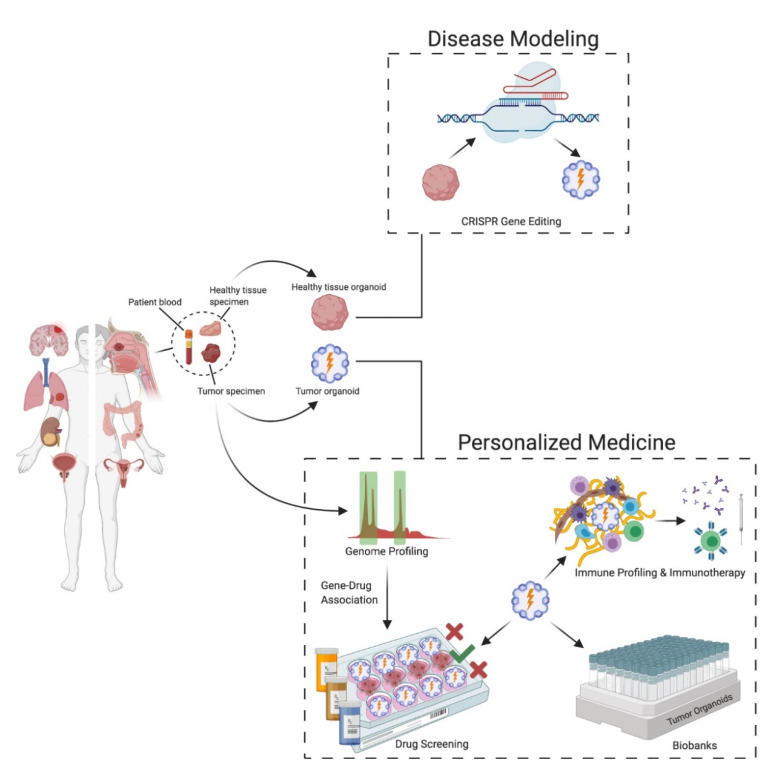
Potential applications of patient-derived organoids (PDOs). PDOs can be derived from surgically resected tumor tissue or tumor biopsy and organoids can also be derived from the normal tissue surrounding the tumor. Gene-editing and screening methods allows the introduction of oncogenic mutations and thus have the potential for studying the tumorigenesis process. Identification of gene–drug associations through genomic profiling permits precision oncology through drug and immunotherapeutic screens. PDOs can be expanded and cryopreserved to establish living organoid biobanks for basic and clinical research purposes.

Tumorigenesis is a complex process involving temporal accumulation of cancer-specific genetic alterations. These genetic alterations have been studied by two different approaches. The top-down approach utilizes transformed cell lines to identify signaling pathways that can affect the oncogenic phenotypes. In the bottom-up approach, one or more mutations are introduced into normal cells on a wild-type background to enable the identification of events associated with cancer initiation, development and progression. However, bottom-up approaches are uncommon due to the limited availability of cell lines from normal or minimally transformed tissues and thus hamper the identification of early oncogenic events. The ability to culture organoids from single cells of normal and diseased tissues combined with tools for editing genomes, epigenomes and transcriptomes provides valuable insights into tumor modeling by both approaches [8,79] (Figure 3). Li et al. reported the multistep modeling of colon, stomach and pancreatic tumors using an epithelial/stromal ALI organoid culture method [49]. In this study, pancreatic organoids were prepared from mice with floxed alleles of *KRAS*, *P53*, *APC* and *SMAD4* and infected with adenovirus expressing Cre-GFP. The oncogene-transformed organoids exhibited marked dysplasia and demonstrated tumorigenicity when transplanted into immunodeficient NOG mice. In 2015, two independent studies attempted to model multistep tumorigenesis in human intestinal organoids using CRISPR/Cas9 gene editing. Matano et al. disrupted tumor suppressors *APC*, *TP53* and *SMAD4* and knocked in *KRASG12V* and *PIK3CAE545* in isogenic wild-type intestinal organoids, and mutant cells were selected based on withdrawal of growth factors in the culture medium. This study demonstrated that the 5 hit organoids grew independent of niche factor supplementation and exhibited well-differentiated adenoma histology upon xenotransplantation [80]. Clever’s group [81] used a similar approach to target *APC*, *TP53*, *SMAD4* and *KRASG12D* in human small intestine and colon organoids. This study demonstrated that loss of *APC* and *P53* were enough for aneuploidy and chromosomal instability. Following these initial reports, several other studies have demonstrated the utility of organoids in the validation and identification of driver mutations in different cancer models [82,83,84]. Although these initial approaches for cancer modeling were low throughput, robust high-throughput-based library screenings are limited owing to technical limitations. Nevertheless, Ringel and colleagues [85] applied genome scale CRISPR screens in wild-type and *APC* mutant intestinal organoids to identify genes involved in TGF-β resistance.

Tumor heterogeneity poses a significant problem for cancer treatment. As organoids can be efficiently generated from single cells, genetic analysis of clonal organoids provides the opportunity to study tumor heterogeneity. When clonal organoids and spheroids were established by multi-region sampling in colon tumors, the number and type of mutations varied per region [86,87]. Intra-tumor heterogeneity was also studied by single-cell transcriptomics in a colon cancer model [88]. Therefore, efficient and long-term culture of organoids allows the study of tumor heterogeneity and evolution. Additionally, organoid models are also useful for studying cancer metastasis, niche factor dependency and stem cells [89].

### 5.2. Personalized Medicine

Cancer heterogeneity causes variability in treatment response among patients. Precision oncology has emerged to address this problem and focuses on creating an individualized treatment plan by identifying prognostic markers and therapeutics. The two major approaches for precision medicine are the inference of the disease behavior using multiomics methods and functionally testing the tumor behavior and response to drugs using personalized cancer medicine models [90]. However, the gene–drug association with targeted therapeutic strategies may be limited due to a lack of understating of tumor response to drugs. Several studies have provided proof-of-concept that tumor organoids recapitulate genomic and histological features of the original tumor from which they were prepared and hence could be utilized for personalized cancer medicine [54,56,91,92,93,94]. Organoid models provide several advantages, including generation of organoids from very small tumor samples such as needle biopsies. Organoids can also be prepared from different regions of the tumor from the same patient. Therefore, tumor organoids have tremendous potential for screening anti-cancer drugs, optimizing immunotherapy and identifying prognostic biomarkers [95] (Figure 3).

### 5.3. Anti-Cancer Drug Screening and Gene–Drug Associations

Use of organoid and spheroid 3D cell culture models for drug screening and drug development applications has gained momentum owing to their resemblance to solid tumors [96,97]. Van de Watering and colleagues cultured organoids from 22 colorectal carcinoma (CRC) patients’ tumor biopsies and from adjacent normal tissue with a success rate of about 90%. They developed a robotized high-throughput drug screening assay in a 384-well format and used a luminescence-based cell viability method as a read out for drug sensitivity [55]. Interestingly, screening of a library of 85 drugs including agents in clinical use and drugs under clinical investigation or currently in a clinical trial resulted in identification of an effective treatment for each patient. Multivariate analysis incorporating IC_50_ values and slopes of the corresponding dose-response curves showed a statistically significant correlation between oncogenic mutations and drug response. Organoids that contained loss of function mutations in *TP53* were resistant to the MDM2 inhibitor nutulin3a and organoids with *KRAS* mutations were resistant to the EGFR inhibitor cetuximab, and therefore, the study demonstrated the feasibility and utility of tumor organoids in high-throughput drug screens, detecting gene–drug associations [55]. 

In another study, Pauli et al. established cell lines, organoids and PDTX from patients with various malignancies. Whole exome sequencing of the tumors detected alterations in known cancer genes in 95.8% of the specimens, of which the majority of them were metastatic advanced disease. When they screened a library of 160 drugs that included both FDA-approved chemotherapeutics and targeted agents under clinical development, there was a high degree of concordance between 2D and 3D cultures. They also identified optimum combinations of drugs for a select set of patients based on genomic alterations. Most importantly, the drugs identified were validated in vivo with matched PDTX models, providing proof-of-concept that the patient-derived organoids can be utilized for complete genomic analysis together with high-throughput drug screening of a comprehensive up-to-date library [98]. Similarly, several other studies have shown that tumor organoids and spheroids can be utilized for anti-cancer drug screening [18,19,21,54,56,92,94,96,99,100,101,102,103,104,105]. 

Recently, few studies have further demonstrated that organoid cultures can be effectively used in predicting response to therapy. Neo-adjuvant therapy (NAT) is becoming a part of standard components of pre-surgical management of locally advanced tumors of several cancer types. However, responses to neo-adjuvant therapy are variable, and hence methods that predict response to NAT are crucial for effective patient care. Yao et al. [106] and Ganesh et al. [107] addressed this issue by demonstrating the response of rectal cancer organoids to NAT. Yao et al. prepared 80 organoids from rectal tumor biopsies of patients recruited in a phase III clinical trial who were treatment-naïve. The patient-derived organoid lines were tested for sensitivity to 5-FU, irinotecan or radiation as single agents using cell viability and organoid size measurements. The most critical element of the study was that the tumor regression scores of the patients after surgery were well-correlated with in vitro organoid response data, with an area under the curve of 0.88 and accuracy of 84% [106]. Ganesh and co-workers cultured organoids from rectal cancer patients and utilized these models to test responses to NAT, including standard chemotherapy regimens or radiation. Most importantly, the responses to NAT in organoids paralleled the patients’ progression-free survival after surgical resection [107]. Similarly, Di-Liello and colleagues utilized a lung cancer spheroid model to show their utility in predicting the response to chemo and immune therapies [108]. These studies represent a significant advance in utilization of organoid cultures as a functional tool in predicting response to treatment and could be leveraged by clinicians to personalize therapies.

Drug-related adverse reactions are major concerns in drug development and assessing the safety profile of drug candidates is indeed a pre-requisite by regulatory bodies for clinical stage development. Drug-related toxicities are routinely performed in cell lines and animal models. Construction of organoids from healthy human kidney, liver and gut, which are main targets of drug-related toxicities, would possibly complement animal toxicology assays. Organoids and spheroids have been constructed from liver, kidney, intestine and heart to model drug-induced toxicities [35,48,109,110,111,112]. Interestingly, organoids constructed from intestines expressed transporters involved in drug influx, efflux and metabolizing cytochrome enzymes, opening the possibility of modeling pharmacokinetics of drugs [113].

### 5.4. Organoid Biobanks

Advancement in long-term culture and cryopreservation methods made it possible to establish repositories of organoids derived from patients. These living biobanks of organoids are characterized genetically and histologically with matched normal organoids. To date, several organoid biobanks have been established since their first report from van de Watering and colleagues in 2015 [55]. Once established, the organoid biobanks can serve as biomaterial for cancer research and personalized medicine. Organoid biobanks are gaining rapid momentum owing to their several advantages. Organoid biobanks are relatively cost-effective and less time-consuming to establish compared to patient-derived xenografts and can be established from needle biopsies and circulating tumor cells [56]. Organoids can be utilized as a pure cell population for DNA sequencing analysis, especially with tumors which yield a low number of cells [114]. Tumor organoids from biobanks were shown to be tumorigenic when xenotransplanted. Most importantly, organoids preserve the genetic and histological features of original tumors. Nevertheless, organoids lose tumor heterogeneity over time in long-term cultures and may acquire new mutations, as shown in microsatellite unstable CRC organoids [93].

### 5.5. Immunotherapy

Evasion of immunity is one of the hallmarks of cancer and effective immunity against tumors is an integral part of many anti-cancer treatments. Immunotherapy has shown encouraging results in numerous cancer types. Multiple reports have demonstrated the potential use of organoid technology in the study of immunotherapy, including interaction of tumors with immune cells, expansion of autologous T cells and assessment of patient response to immune checkpoint inhibition [115]. One report demonstrated the ability of expanding autologous T cells by co-culturing matched PBMC with PDOs from colorectal and non-small cell lung cancer patients. The PDOs generated from these patients demonstrated heterogeneity and retained the genetic and histological features of the original tumor. The enriched T cells were used to model the efficiency of matched organoid killing [116]. Several organoid models including chondroma, glioblastoma, melanoma and colorectal carcinoma, following two organoid approaches, have been developed to study responses to immune checkpoint blockade [45,117,118]. In the first approach, established tumor organoids were co-cultured with autologous immune cells. Tumor cell viability or granzyme production was measured in these short-term cultures as a read out for the cytotoxic effect of T cells upon organoid and immune cell co-culture in the presence of immune checkpoint blockade. Votanopoulas et al. developed organoids from matched melanoma and lymph node tissue from the same patient as symbiotic tumor/immune organoids. The success rate of this tumor/node organoid model was about 90%, and more interestingly, the response of these organoids to immune checkpoint inhibitors correlated strongly with clinical responses of the patients (85%) [119]. The second approach aimed to generate tumor organoids that preserved endogenous immune and stromal cells, thus mimicking the native tumor microenvironment. In 2018, Neal et al. constructed PDOs from greater than 100 patient biopsies using the ALI method. PDOs generated by the ALI method recapitulated the tumor microenvironment, preserved the TCR repertoire and T cells were shown to respond to immune checkpoint inhibition [58]. In the same year, Jenkins et al. constructed organotypic tumor spheroids in collagen hydrogels in a 3D microfluidic device. These organoids retained native immune cells [75,76]. Yet, other studies have shown that organoids can also be generated from tumors with low mutational burden and can be targeted by CAR-T cells or CAR-NK-92 cells [120]. Della Corte et al. demonstrated the sensitivity of programmed death-ligand 1 (PD-L1) inhibition in combination with MEK-targeted inhibitor MEK-I in a lung cancer spheroid model [20]. Taken together, these studies demonstrate that organoids and spheroids can be established from patients with various tumor types for modeling immunotherapy. 

## 6. Conclusions

In this review, we have summarized spheroid and organoid culture methods and their potential applications in cancer modeling, drug screening and personalized medicine. Although 3D cell culture models have been described for about half a century, the past decade has seen tremendous enthusiasm in organoid culture methods. As demonstrated in recent publications, generation of organoids from different cancer types has proven to be an outstanding model for the interrogation of different phases of cancer biology and drug discovery [121]. Organoids from living biobanks serve as a crucial source of biomaterial for world-wide use and will be an oasis for rare cancer types. Static models of both spheroids and organoids are not perfused with nutrient-rich medium flowing through the vasculature and hence lack the tissue–tissue interaction. Organoids on microfluidic devices or tumor-on-chips offer several advantages that could help fill this gap. These include the study of recruitment of circulating immune cells as well as physiological dosing of therapeutics. Extracellular matrices popularly used in organoid cultures are originated from animal sources known to have batch-to-batch variability in their composition and may hamper the reproducibility of experiments. Progress is being made to prepare clinical-grade collagen as well as synthetic materials such as polyethylene glycol with defined and consistent compositions. Unified organoid culture methods that support efficient generation of organoids from different cancer types are very much needed. Efficient and cost-effective establishment of tumor-derived organoids in a clinically relevant timeframe would enable drug screening for individual cancer patients. Optimization of methods for robustness and sensitivity in drug screening with PDO’s coupled with validation of drug response predictions from clinical studies would enable the implementation of organoid-based personalized medicine in the clinic. Generating organoids from both healthy and diseased tissues holds great promise for a positive impact on cancer research and drug development. 

## Figures and Tables

**Table 1:** Pre-clinical models in cancer research.

Model	Advantages	Disadvantages
**Cell lines (two-dimensional (2D) cell culture)**	Reproducible and rapid growth Low-cost and simple high-throughput drug screening and toxicity testing	Oversimplified model of cancer Low success rate of establishment for some tumor types Lack of tumor heterogeneity and tumor microenvironment (TME)
**Syngeneic transplantation**	Reproducible and rapid growth of tumor No host breeding requirements Use of orthotopic models	Relatively few transplantable cell lines Limited host strains Lack of tumor heterogeneity
**Patient-DerivedTumor Xenografts (PDTX)**	Recapitulate human disease including tumor heterogeneity and cell types Partly recapitulates TME Anti-tumor responses may be comparable to patient responses. Ability to study metastasis	Requires immune-deficient hosts Relies on immune cells transferred with xenograft or requires reconstitution of human immune system (short term) Low tumor implantation rates
**Three-dimensional (3D) cell culture**	Bridges the gap between 2D cell cultures and in vivo models Recapitulate tumors histologically and genetically Retains tumor heterogeneity	Relatively costly and time-consuming Unified methods of organoid production and tools to analyze them are limited Current organoid models (with few exceptions) do not reconstitute the complex TME and the methods that are capable can do so only for a short period

**Table 2:** Comparison of spheroids and organoid models.

Features	Spheroids	Organoids
**Cellular source**	Cell lines, multicellular mixtures, primary cells, tumor cells and tissues	Embryonic stem cells, adult stem cells or induced pluripotent cells, tumor cells and tissues
**3D organization**	Self-assembly involving cell–cell aggregation and adhesion. Self-organization occurs in certain spheroids models	Self-organization and self-assembly involving differentiation of cells in response to physical and chemical cues, forming a complex structure
**Organ physiology**	Layers of heterogenous cells proliferating, quiescent and necrotic cells, and transiently resembles 3D cellular organization	Different cell lineages that reflect the structure and function of the organ, at least in part
**3D culture conditions**	Cultured with or without extracellular matrix and growth factors	Requires extracellular matrix and a cocktail of growth factors
